# Supplementary material for: Mechanistic insight into the role of AUXIN RESISTANCE4 in trafficking of AUXIN1 and LIKE AUX1-2
Source: Plant Physiol. 2023 Sep 30;194(1):422–33. doi: 10.1093/plphys/kiad506 (PMC10756756; doi:10.1093/plphys/kiad506)
Supplement: kiad506_Supplementary_Data [file kiad506_supplementary_data.zip › Suppemental Dataset S1/5445e4f6eaa2385e-AtAXR4/dom_report.pdf]

Phyre2

Email	charlie.hodgman@nottinghamac.uk
Description	AtAXR4___
Date	Tue Jan 10 14:09:39 GMT 2023
Unique Job ID	5445e4f6eaa2385e

Domain analysis

Rank	Aligned region
1	c6g75B_
2	c2vf2A_
3	c4mj3B_
4	d1bn7a_
5	c3i28A_
6	c1cr6A_
7	c6y9gB_
8	c4ns4A_
9	d1mj5a_
10	c3u1tA_
11	c4c6hA_
12	c3a2nF_
13	c3wibB_
14	c7jqzE_
15	d1cr6a2
16	d1zd3a2
17	c2e3jA_
18	c7avrG_
19	c2qvbA_
20	c5xmdA_
21	
22	
23	
24	
25	
26	
27	
28	
29	
30	
31	
32	
33	
34	
35	
36	
37	
38	
39	
40	
41	
42	
43	
44	
45	
46	
47	
48	
49	
50	
51	
52	
53	
54	
55	
56	
57	
58	

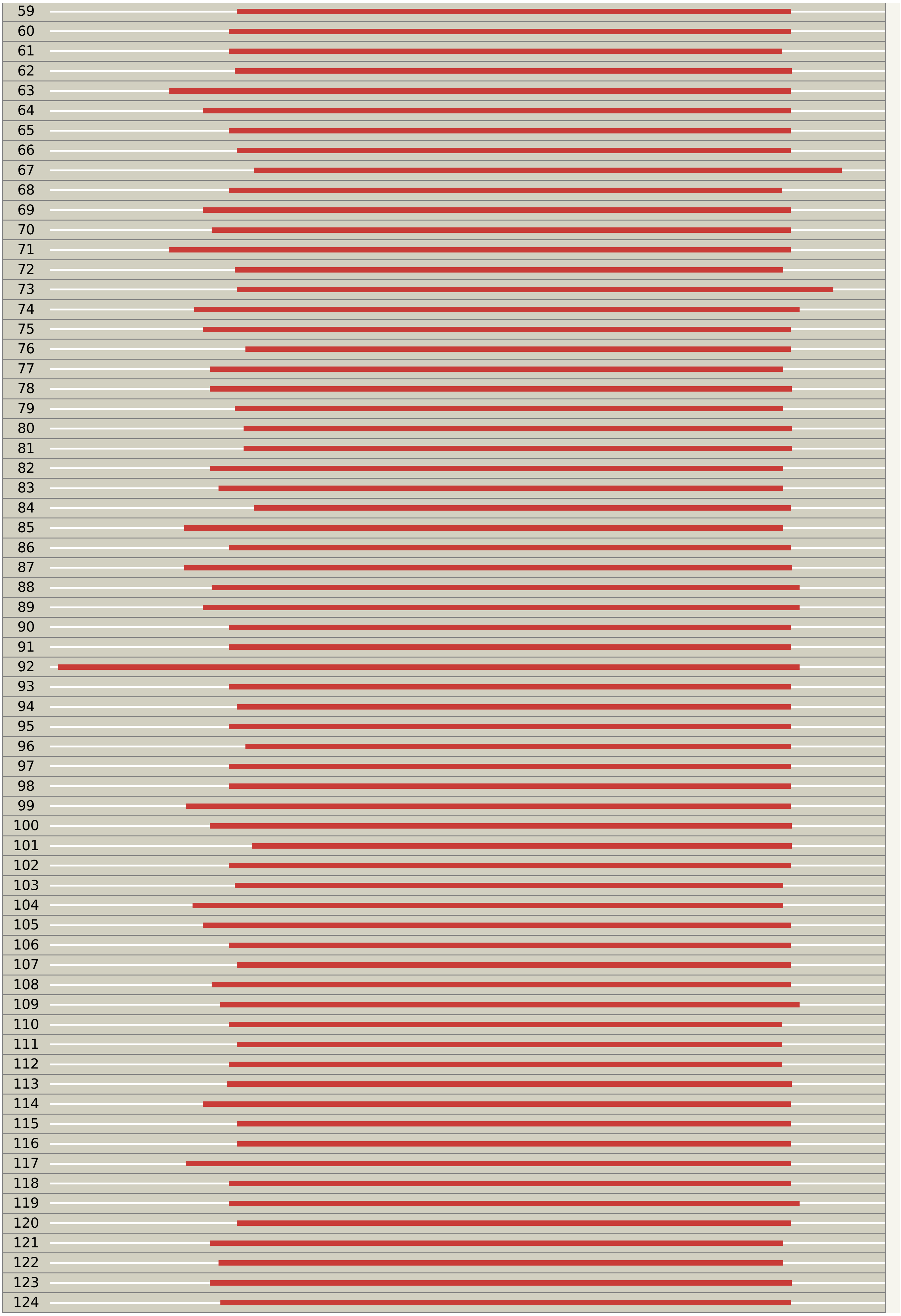
59
60
61
62
63
64
65
66
67
68
69
70
71
72
73
74
75
76
77
78
79
80
81
82
83
84
85
86
87
88
89
90
91
92
93
94
95
96
97
98
99
100
101
102
103
104
105
106
107
108
109
110
111
112
113
114
115
116
117
118
119
120
121
122
123
124

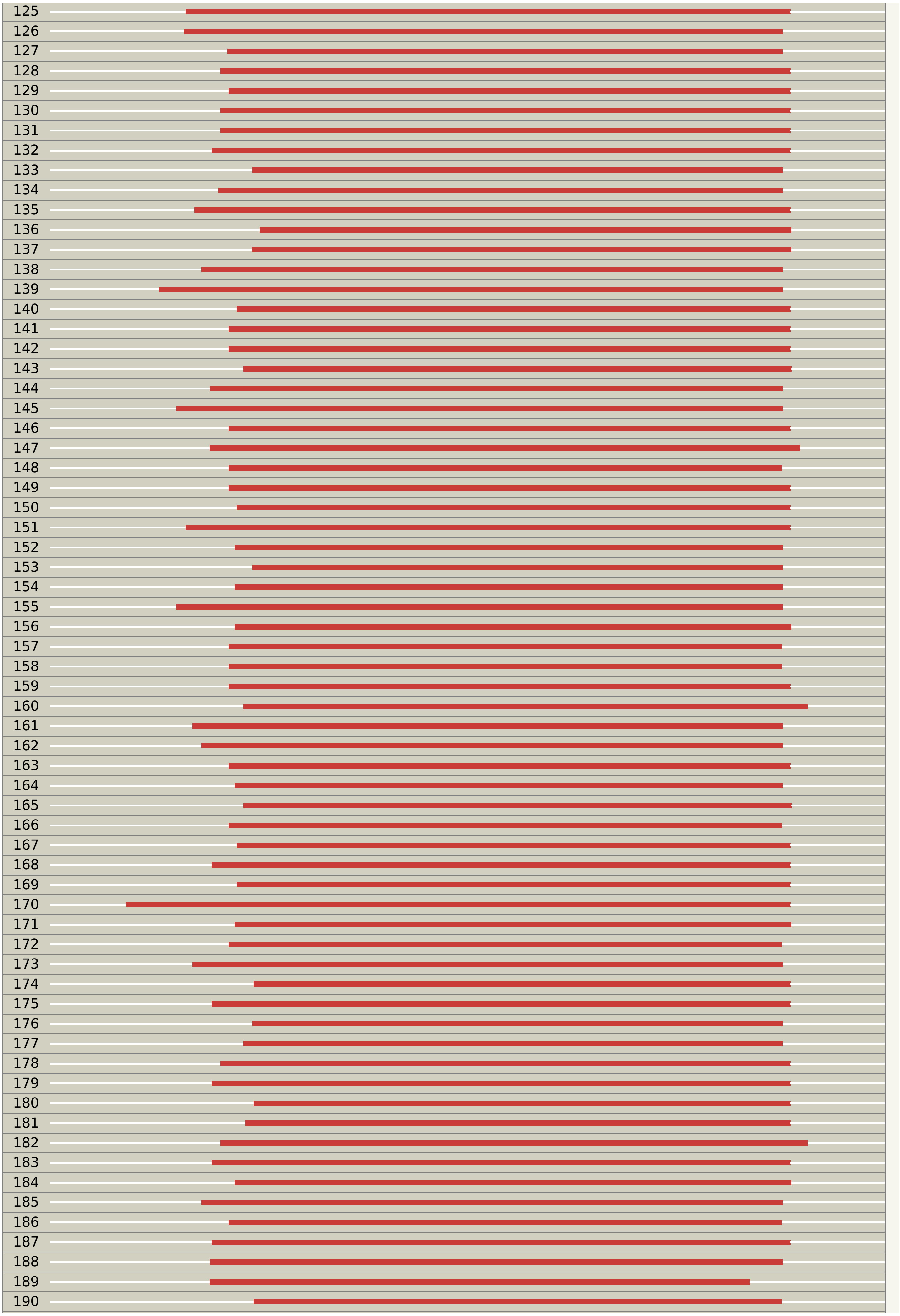
125
126
127
128
129
130
131
132
133
134
135
136
137
138
139
140
141
142
143
144
145
146
147
148
149
150
151
152
153
154
155
156
157
158
159
160
161
162
163
164
165
166
167
168
169
170
171
172
173
174
175
176
177
178
179
180
181
182
183
184
185
186
187
188
189
190

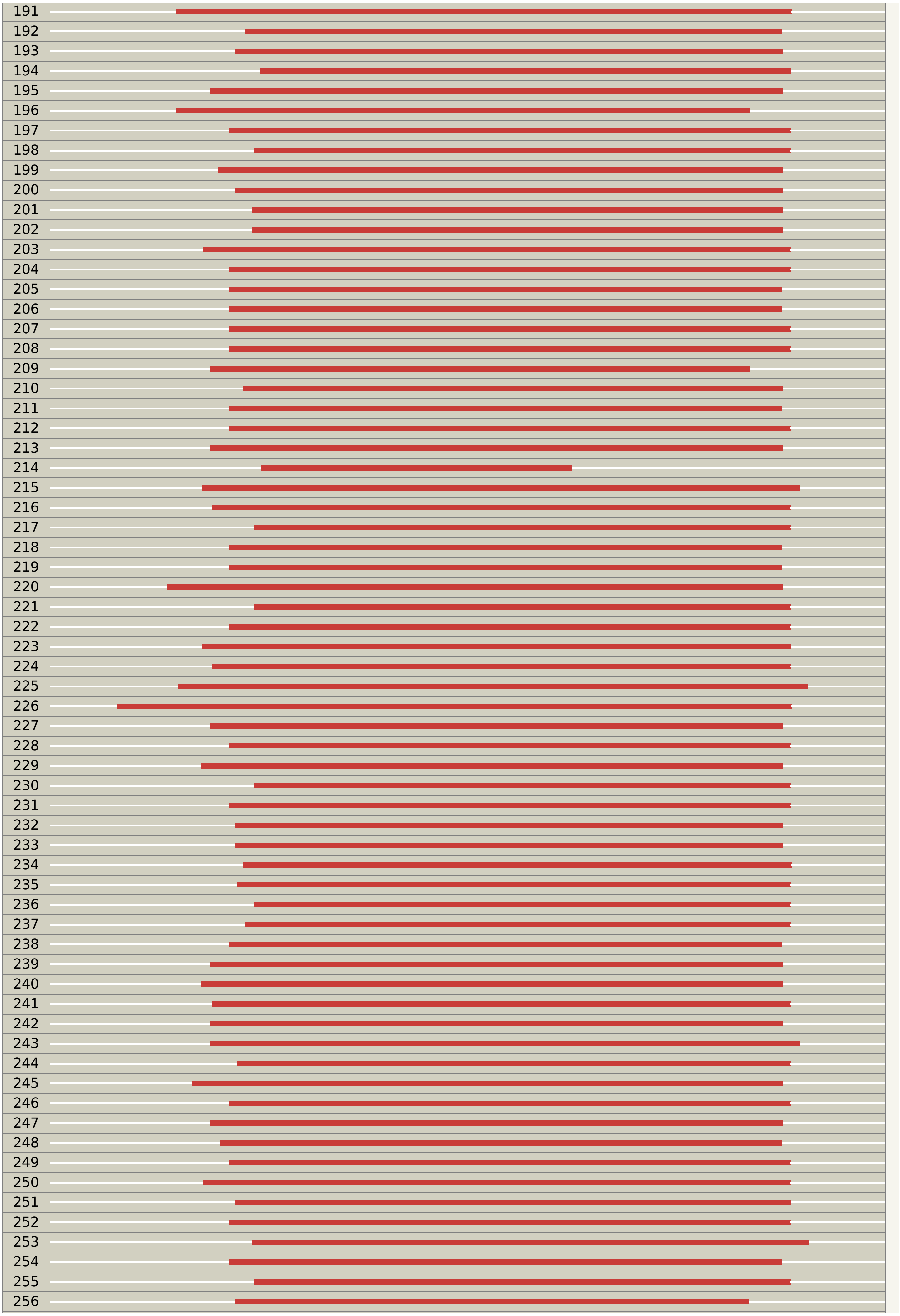
191
192
193
194
195
196
197
198
199
200
201
202
203
204
205
206
207
208
209
210
211
212
213
214
215
216
217
218
219
220
221
222
223
224
225
226
227
228
229
230
231
232
233
234
235
236
237
238
239
240
241
242
243
244
245
246
247
248
249
250
251
252
253
254
255
256

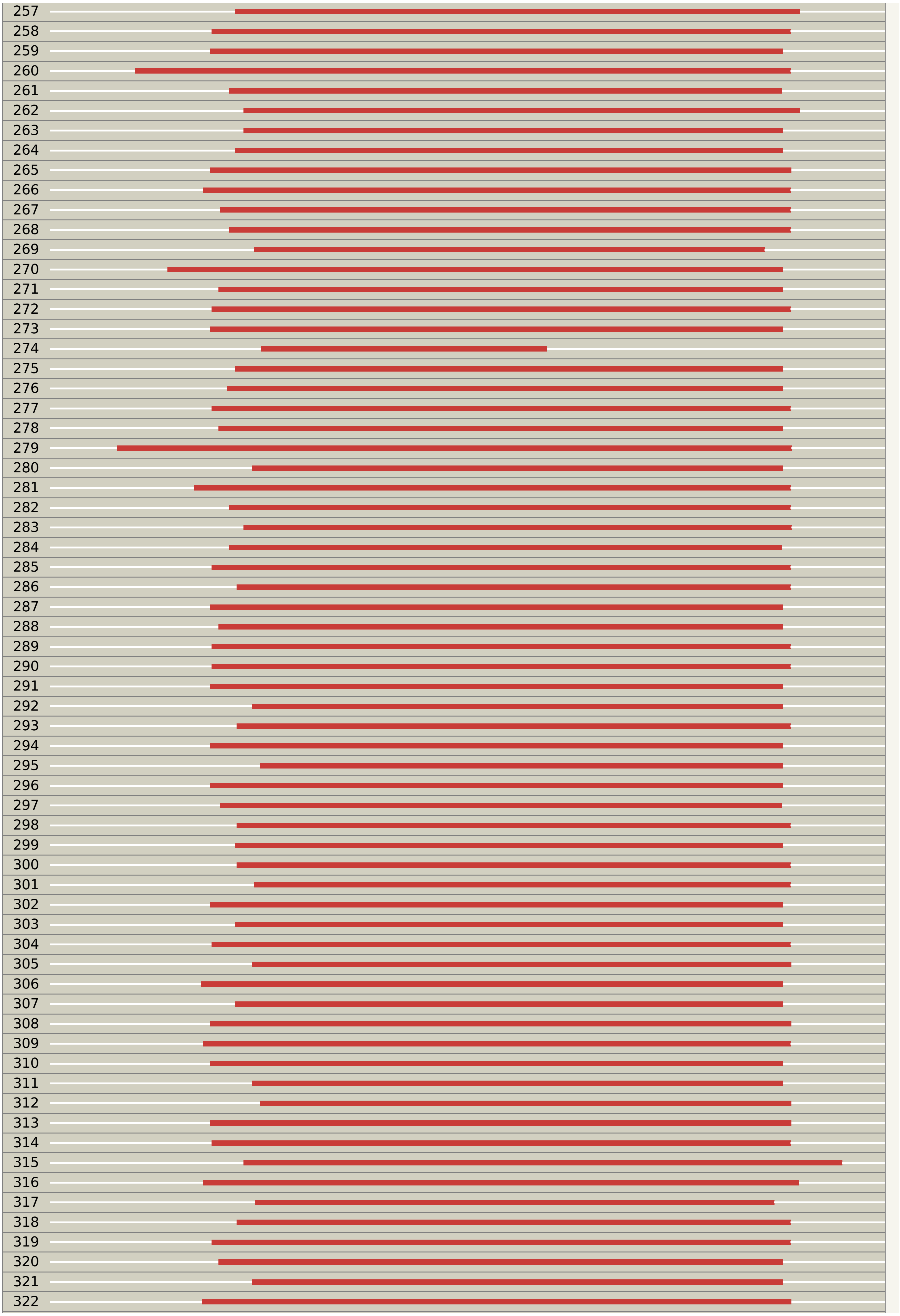
257
258
259
260
261
262
263
264
265
266
267
268
269
270
271
272
273
274
275
276
277
278
279
280
281
282
283
284
285
286
287
288
289
290
291
292
293
294
295
296
297
298
299
300
301
302
303
304
305
306
307
308
309
310
311
312
313
314
315
316
317
318
319
320
321
322

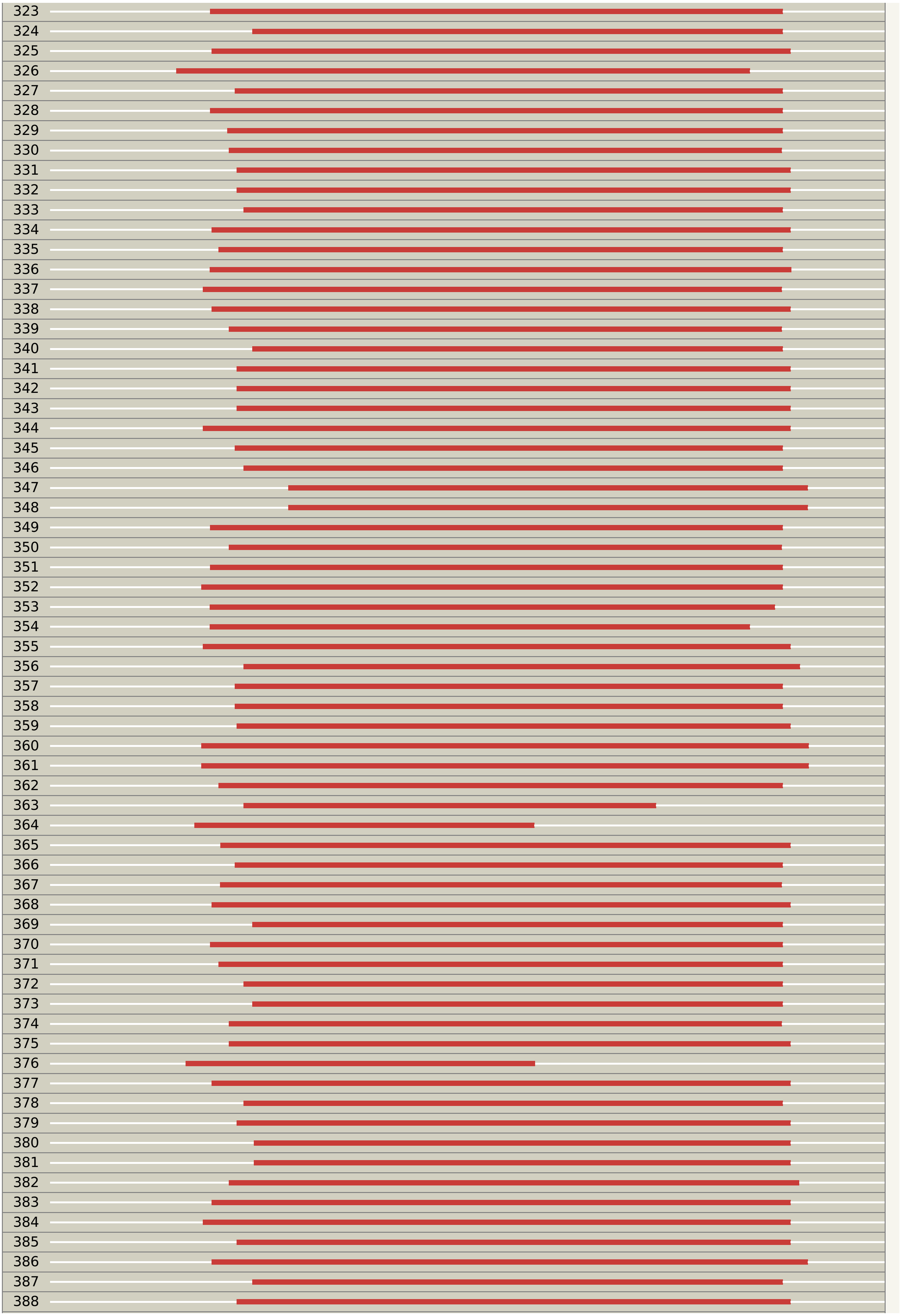
323
324
325
326
327
328
329
330
331
332
333
334
335
336
337
338
339
340
341
342
343
344
345
346
347
348
349
350
351
352
353
354
355
356
357
358
359
360
361
362
363
364
365
366
367
368
369
370
371
372
373
374
375
376
377
378
379
380
381
382
383
384
385
386
387
388

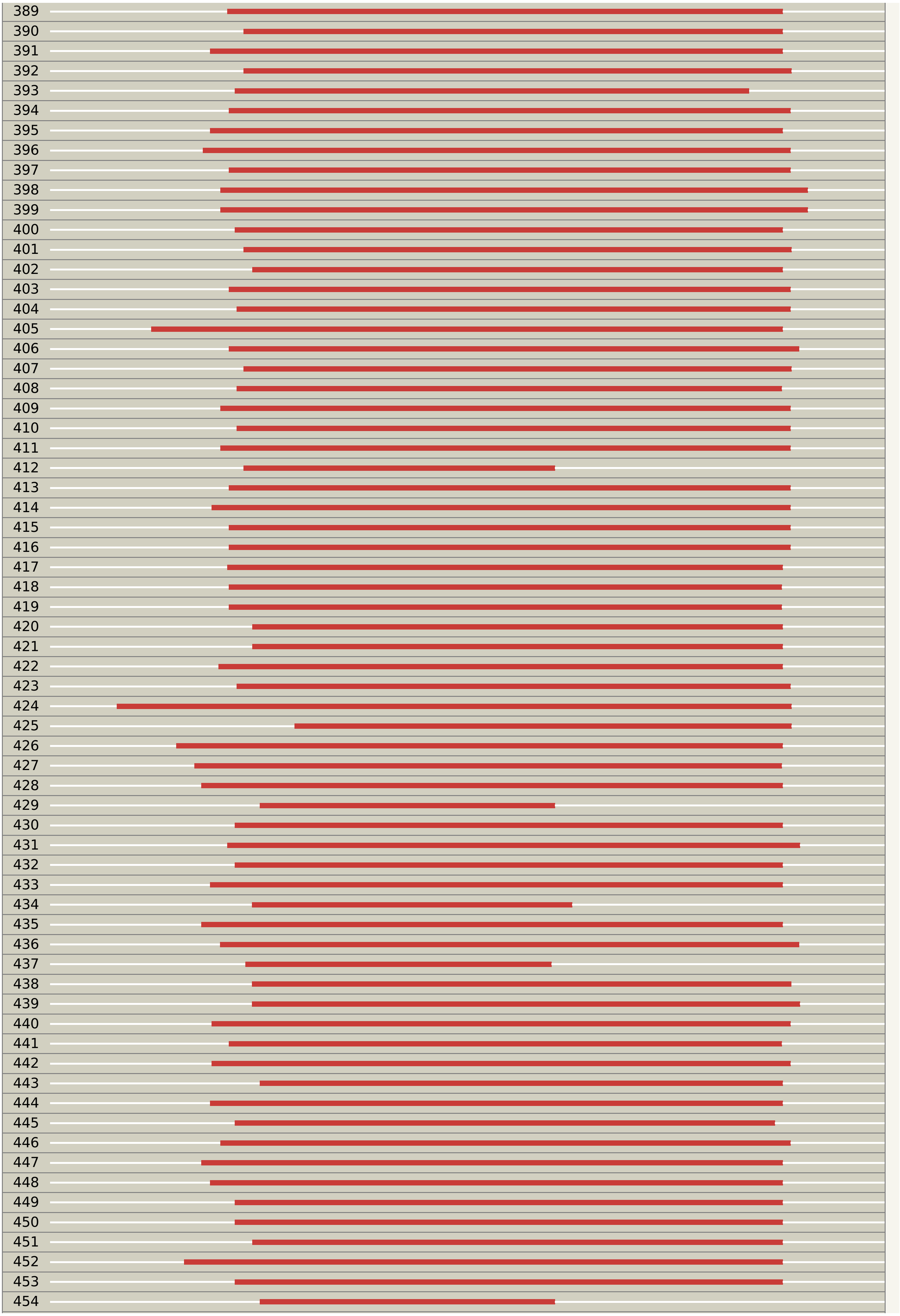
389
390
391
392
393
394
395
396
397
398
399
400
401
402
403
404
405
406
407
408
409
410
411
412
413
414
415
416
417
418
419
420
421
422
423
424
425
426
427
428
429
430
431
432
433
434
435
436
437
438
439
440
441
442
443
444
445
446
447
448
449
450
451
452
453
454

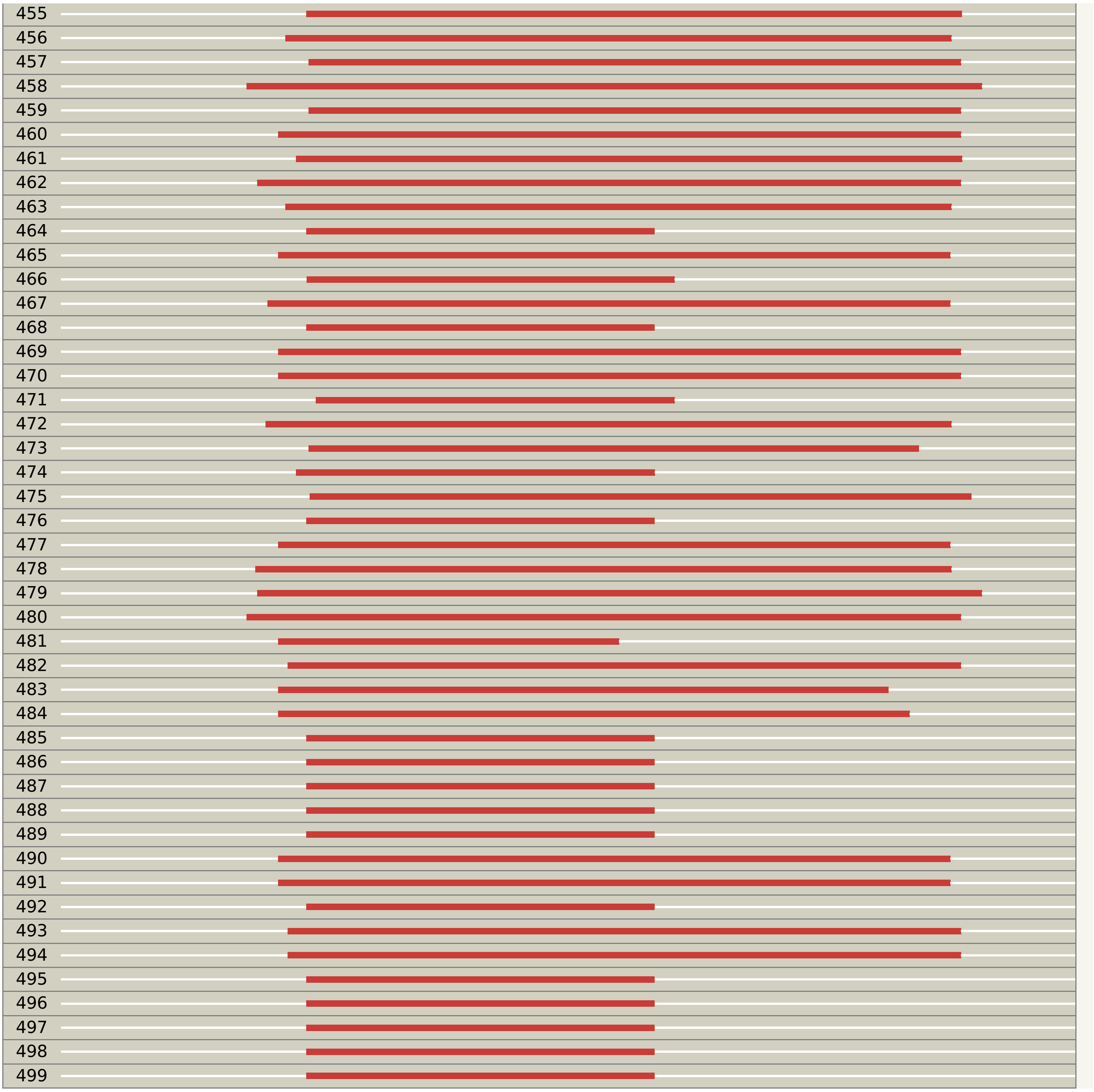
455
456
457
458
459
460
461
462
463
464
465
466
467
468
469
470
471
472
473
474
475
476
477
478
479
480
481
482
483
484
485
486
487
488
489
490
491
492
493
494
495
496
497
498
499
